# Contributions of Myosin Light Chain Kinase to Regulation of Epithelial Paracellular Permeability and Mucosal Homeostasis

**DOI:** 10.3390/ijms21030993

**Published:** 2020-02-03

**Authors:** Wei-Qi He, Jing Wang, Jian-Ying Sheng, Juan-Min Zha, W. Vallen Graham, Jerrold R. Turner

**Affiliations:** 1 Jiangsu Key Laboratory of Neuropsychiatric Diseases and Cambridge-Suda (CAM-SU) Genomic Resource Center, Medical College of Soochow University, Suzhou 215123, China; 20184250017@stu.suda.edu.cn (J.W.); 20184250012@stu.suda.edu.cn (J.-Y.S.); zhajuanmin@suda.edu.cn (J.-M.Z.); 2 Thelium Therapeutics, New York, NY 10014, USA; 3 Laboratory of Chemical Biology & Signal Transduction, The Rockefeller University, New York, NY 10065, USA; 4 Laboratory of Mucosal Barrier Pathobiology, Department of Pathology, Brigham and Women’s Hospital and Harvard Medical School, Boston, MA 02115, USA

**Keywords:** tight junction, barrier function, inflammatory bowel disease, drug development, mucosal immunology, cytokines, ZO-1, occludin, claudin, actomyosin

## Abstract

Intestinal barrier function is required for the maintenance of mucosal homeostasis. Barrier dysfunction is thought to promote progression of both intestinal and systemic diseases. In many cases, this barrier loss reflects increased permeability of the paracellular tight junction as a consequence of myosin light chain kinase (MLCK) activation and myosin II regulatory light chain (MLC) phosphorylation. Although some details about MLCK activation remain to be defined, it is clear that this triggers perijunctional actomyosin ring (PAMR) contraction that leads to molecular reorganization of tight junction structure and composition, including occludin endocytosis. In disease states, this process can be triggered by pro-inflammatory cytokines including tumor necrosis factor-α (TNF), interleukin-1β (IL-1β), and several related molecules. Of these, TNF has been studied in the greatest detail and is known to activate long MLCK transcription, expression, enzymatic activity, and recruitment to the PAMR. Unfortunately, toxicities associated with inhibition of MLCK expression or enzymatic activity make these unsuitable as therapeutic targets. Recent work has, however, identified a small molecule that prevents MLCK1 recruitment to the PAMR without inhibiting enzymatic function. This small molecule, termed Divertin, restores barrier function after TNF-induced barrier loss and prevents disease progression in experimental chronic inflammatory bowel disease.

## 1. Structure of Epithelial Intercellular Junctions

Intestinal mucosal surfaces are covered by a single layer of columnar epithelial cells required for absorptive and defensive functions. This requires cellular polarization in order to ensure appropriately-oriented membrane specializations, protein and lipid trafficking, and vectorial transport of ions and larger solutes [1,2,3]. For example, the dense forests of microvilli that increase apical surface area and facilitate nutrition absorption [4] are not needed on the basolateral surface, which interacts with adjacent epithelial cells, immune cells, and the basement membrane.

**Figure 1:**
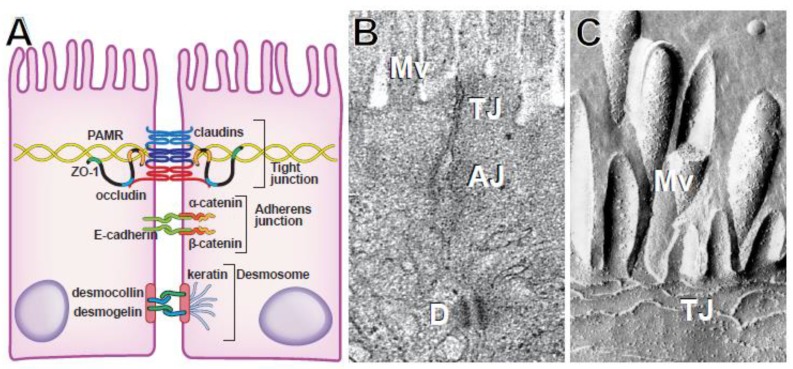
The structure of epithelial intercellular junctions. (**A**) Schematic showing interactions between the perijunctional actomyosin ring (PAMR), zonula occludens-1 (ZO-1), occludin, claudins at the tight junction; E-cadherin, α-catenin, and β-catenin at the adherens junction; and desmogelin, desmocollin, and intermediate filaments at the desmosome. (**B**) Transmission electron micrograph showing the tight junction (TJ), adherens junction (AJ), desmosome (D), and microvilli (Mv). From Turner. Nat Rev Immunol 2009. (**C**) Freeze-fracture electron micrograph of intramembranous tight junction strands. From Shen et al. *Annu. Rev. Physiol.* 2011.

In order to maintain polarized function and regulate the stimuli to which apical and basolateral surfaces are exposed, intestinal epithelial cells must establish a physical barrier that prevents free diffusion across the paracellular shunt pathway [5,6,7,8,9,10,11,12,13,14]. This barrier is formed by a series of junctions that provide different types of intercellular connections. This apical junctional complex cannot be resolved by light microscopy but can be seen as the terminal bar, a dense spot or bar where apical and lateral membranes meet [5,15,16,17]. Transmission electron microscopy allows visualization of distinct regions such as, from apical to basal, tight junctions (zonulae occludens), adherens junctions (zonulae adherens), and desmosomes [5] (Figure 1A,B). Tight junctions are sites of close apposition of adjacent cell membranes, termed kiss sites, where the adjacent plasma membrane outer leaflets appear to fuse into a single layer. This corresponds to the site at which paracellular flux of macromolecular probes is blocked. Although some microfilaments are associated with tight junctions, they are most concentrated at adherens junctions [18,19,20], which do not form barriers to paracellular flux but are critical to intercellular adhesion and maintenance of the apical junctional complex. Finally, desmosomes can be recognized as electron dense membrane structures associated with intermediate, i.e., cytokeratin, filaments [21,22,23,24].

Tight junction structure is far more interesting when viewed by freeze-fracture electron microscopy [25,26,27]. This reveals an anastomosing, mesh-like network of intramembranous strands (Figure 1C). Closer examination shows that the strands are composed of individual particles, causing some observers to compare the appearance to a string of pearls. The particles are thought to represent tight junction protein complexes that include polymers of claudin family proteins [7,28,29,30]. Consistent with this, alterations in the ensemble of claudin proteins expressed can modify the architecture of the strand network [31]. Although lipids must also be associated with tight junction structures, these are less well-characterized. It is, however, known that tight junctions are cholesterol- and sphingolipid-rich microdomains and that cholesterol depletion reduces both strand network complexity and paracellular barrier function [32,33,34].

## 2. The Paracellular “Shunt” Pathway

**Figure 2:**
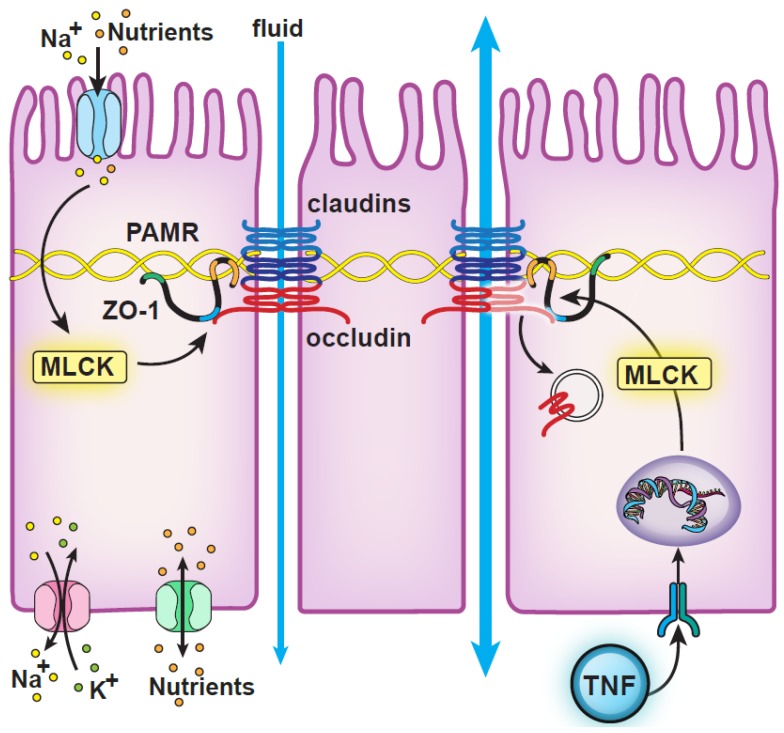
Roles of myosin light chain kinase (MLCK) in physiological and pathophysiological tight junction regulation. PAMR: perijunctional actomyosin ring; TNF: tumor necrosis factor.

The intestinal mucosa confines potentially injurious contents within the lumen. The paracellular barrier, however, cannot be absolute; it must be selectively permeable to water, ions, small nutrients, and selected macromolecules in order to facilitate passive transport that is essential for nutrition and metabolism. Permeability of tight junction flux pathways must, therefore, be precisely regulated. For example, tight junction permeability is increased during nutrient absorption. This is triggered by Na^+^–nutrient cotransport, which increases paracellular permeability by activating myosin light chain kinase (MLCK) to cause perijunctional actomyosin ring (PAMR) remodeling [35,36,37,38,39] (Figure 2). In the context of nutrient absorption, these permeability increases are limited to small, nutrient-sized molecules [35,40]. This couples with the transepithelial gradients established by active, transcellular transport, i.e., Na^+^ and nutrient release into the basal extracellular milieu, to drive passive paracellular fluid absorption [37,41,42]. The absorbed fluid, from the unstirred layer, which contains high concentrations of nutrient monomers as a consequence of brush border hydrolase, e.g., disaccharidase and peptidase, activity [43,44]. Fluid absorption therefore carries nutrients, against their concentration gradient, by the mechanisms of solvent drag [42,44,45]. Increased tight junction permeability amplifies this process and allows total transepithelial nutrient absorption to exceed the maximum capacity of transcellular transport pathways [37,38,41,45,46,47,48]. A similar process allows claudin-2-mediated paracellular Na^+^ transport to complement transcellular Na^+^ transport and enhance the efficiency of Na^+^ reabsorption in the renal proximal tubule [49].

In contrast to Na^+^–nutrient cotransport [35,40], MLCK activation by inflammatory stimuli, e.g., tumor necrosis factor α (TNF), increases paracellular permeability to larger macromolecules, up to ~125 Å in diameter, thereby activating the low capacity leak pathway [50,51,52,53,54,55] (Figure 2). The differences between these two forms of MLCK-dependent barrier regulation are incompletely understood, but it is notable that occludin endocytosis occurs in response to TNF but not Na^+^–nutrient cotransport (Figure 2).

Some claudin proteins, e.g., claudin-2, form actively-gated paracellular channels that define the pore pathway [52,53,56] In contrast to the leak pathway, the high capacity pore pathway channels are exquisitely size- and charge-selective, with a cutoff of ~8 Å diameter [57,58]. This limits the pore pathway to small ions and water and is too small to accommodate even small nutrients, e.g., glucose and amino acids. The pore pathway is, however, essential for nutrient transport as it allows Na^+^ ions within the lamina propria, i.e., beneath the epithelial cells, to leak back into the gut lumen [59,60]. This provides the lumenal Na^+^ that is required for Na^+^–nutrient cotransport, the dominant route of intestinal nutrient absorption. Thus, mice lacking the two principal claudins that form paracellular cation channels within the intestinal epithelium die in the first few weeks of life as a result of nutrient malabsorption [59]. The remainder of this review will focus on the leak pathway. Claudin channels and the pore pathway are discussed elsewhere [61,62,63,64].

Na^+^–nutrient cotransport at the apical brush border activates MLCK. Nutrients and Na^+^ exit across the basolateral membrane via diffusive exchangers and the Na^+^/K^+-^ATPase, respectively. Although not indicated here, activation of other transporters, e.g., apical NHE3-mediated Na^+^ absorption, further increases basolateral Na^+^ [65,66,67,68,69]. Together, these events increase lamina propria osmolarity [70] to draw fluid across the tight junction. The modest, size-selective, increases in leak pathway permeability elicited by MLCK allow small nutrient-sized molecules to be carried with this fluid via solvent drag [44]. The end result is amplification of transcellular nutrient absorption by paracellular water and nutrient absorption. MLCK is also activated by tumor necrosis factor (TNF), but, in this case, myosin light chain phosphorylation triggers caveolar endocytosis of occludin that causes much greater increases in macromolecular permeability. Although not shown here, TNF also inhibits apical NHE3-mediated Na^+^ absorption, in part explaining why fluid secretion accompanies TNF-induced increases in paracellular permeability [71].

## 3. MLCK, ZO-1, and Occludin Regulate the Leak Pathway

### 3.1. MLCK Regulates Leak Pathway Permeability

Morphological analyses of rodent mucosae identified the appearance of intrajunctional dilatations and PAMR condensation as the morphological correlates of increased paracellular permeability induced by Na^+^–nutrient cotransport [37,38,39]. In vitro models were then used to show that MLCK-mediated phosphorylation of myosin II regulatory light chain (MLC) was required for paracellular permeability increases that follow activation of Na^+^–nutrient cotransport [35], enteropathogenic E. coli infection [72], or TNF stimulation [50]. MLCK inhibition also prevented Na^+^–nutrient cotransport-induced paracellular permeability increases in rodent mucosae [35] and was associated with Na^+^–nutrient cotransport-induced permeability increases in human intestine [36]. Finally, transgenic expression of constitutive-active MLCK increased paracellular permeability in vitro [73,74] and in vivo [75]. MLCK is, therefore, a key signaling node in physiological and pathophysiological regulation of epithelial tight junctions.

### 3.2. MLCK Regulates Tight Junction Protein Interactions and Structure

The importance of MLCK to tight junction regulation in vivo was initially demonstrated in the context of acute, systemic T cell activation [51]. Administration of T cell-activating anti-CD3 antibodies to mice and humans induces a cytokine storm that includes massive systemic release of TNF [76,77,78]. This results in an acute, self-limited, TNF-dependent diarrhea [79,80]. Ultrastructural examination revealed PAMR condensation similar to that induced by Na^+^–nutrient cotransport; intrajunctional dilatations were not detected and are likely a consequence of the massive paracellular water absorption driven by Na^+^–nutrient cotransport [51]. Further study confirmed increased intestinal epithelial MLC phosphorylation following T cell activation. Notably, the peak of epithelial MLC phosphorylation coincided with maximal intestinal fluid accumulation, and both MLC phosphorylation and fluid accumulation resolved with similar time courses [51]. Analysis of tight junction protein distributions by immunofluorescence microscopy demonstrated subtle changes in ZO-1 localization that included reduced ZO-1 staining and increased waviness of bicellular tight junction profiles within intestinal epithelia of anti-CD3-treated mice [51]. Genetic MLCK activation in vitro also induced undulations within ZO-1-labeled tight junctions [74]. Enzymatic MLCK inhibition reversed these changes, both in vivo and in vitro [51,74]. It may be that these morphological alterations reflect changes in ZO-1 and ZO-2 phase separation [81].

In vitro ZO-1 reorganization induced by MLCK activation was accompanied by similar changes in occludin and F-actin profiles, and these proteins continued to be closely associated [74]. In contrast, claudins 1 and 2 appeared to remain at tight junctions at sites of ZO-1, occludin, and F-actin invaginations [74]. Consistent with this, a previous ultrastructural study of rodent mucosae demonstrated ZO-1 displacement from junctional fibrils, which we now understand to be comprised of claudin polymers, in the context of Na^+^–nutrient cotransport-induced increases in paracellular permeability [82]. Thus, structural changes induced by MLCK-dependent MLC phosphorylation include reorganization of tight junction protein complexes.

Recognition that tight junction protein complexes undergo continuous remodeling, even at steady-state, i.e., in the absence of exogenous stimuli [83], led to the hypothesis that MLCK-mediated leak pathway regulation might be a consequence of altered remodeling dynamics. In vitro MLCK inhibition had no effect on anchoring and exchange of claudin-1, occludin, or F-actin and epithelial tight junctions [84]. In contrast, ZO-1 exchange was markedly reduced following MLCK inhibition [84]. Remarkably, enzymatic MLCK inhibition also reduced ZO-1 exchange in vivo [84]. This increase in ZO-1 anchoring at the tight junction following MLCK inhibition was mapped to the actin binding region (ABR) of ZO-1 [84]. Mutation of ZO-1 to delete the ABR caused a modest increase in basal mobile fraction but rendered ZO-1 insensitive to MLCK inhibition. Moreover, the free ABR was able to act as a dominant negative inhibitor of MLCK-mediated barrier regulation [84]. These data suggest that one mechanism by which MLCK regulates tight junction structure and function involves interactions mediated by the ZO-1 ABR.

### 3.3. MLCK Activation Triggers Tight-Junction Protein Endocytosis

In addition to subtle ZO-1 reorganization, TNF induced endocytosis of the tight junction protein occludin in vitro and in vivo [51,54,85] (Figure 2). Although TNF also triggered endocytosis of other tight junction proteins in vitro, only occludin was internalized in vivo. This was, initially, difficult to understand, as intestinal barrier function in occludin knockout mice has been reported to be similar to that of wildtype mice [86,87]. MLCK-dependent occludin endocytosis was, however, the first change that accompanied actin depolymerization-induced barrier loss in vitro [88]. This internalization occurred via caveolae, and inhibition of caveolar endocytosis prevented actin depolymerization-induced barrier loss in vitro [88]. MLCK inhibition blocked the caveolin-1 dependent endocytosis of occludin.

Further investigation showed that occludin endocytosis triggered by TNF and related cytokines was mediated by caveolae, both in vitro and in vivo [85,89,90]. Moreover, inhibition of caveolar endocytosis blocked this inflammation-induced barrier loss. Most strikingly, neither occludin endocytosis nor barrier loss occurred after TNF treatment of caveolin-1 knockout mice [85]. This does not, however, demonstrate that occludin endocytosis is essential for TNF-induced barrier loss; it only shows that occludin is a reliable marker of the caveolar endocytosis that drives such barrier loss. To determine the specific contribution(s) of occludin to TNF-induced barrier loss, responses of transgenic mice expressing enhanced green fluorescent protein (EGFP)-occludin within intestinal epithelial cells were analyzed. TNF did not induce diarrhea, i.e., net fluid secretion, in these occludin overexpressing mice [85]. Barrier loss was also markedly attenuated in EGFP-occludin transgenic mice [85]. Thus, removal of occludin from the tight junction is required for TNF-induced increases in tight junction permeability.

### 3.4. Interactions Mediated by the Occludin OCEL Domain Regulate Leak Pathway Barrier Function

Despite the reported absence of barrier dysfunction in occludin knockout mice, several studies have shown that occludin knockdown in vitro increases paracellular permeability to macromolecules [89,91]. Similarly, acute occludin downregulation by miR-122a transfection in vivo increased paracellular permeability to 10 kDa dextran [92]. It may, therefore, be that compensation by other members of the tight junction associated Marvel protein (TAMP) family [93,94,95,96,97,98] supports normal macromolecular barrier function in occludin knockout mice.

Detailed analyses of Caco-2 intestinal epithelial cell monolayers demonstrated that occludin knockdown specifically increased paracellular permeability by a pathway with a theoretical diameter of 125 Å, presumably the leak pathway [89]. Similar studies of occludin-knockdown MDCK cell monolayers demonstrated increased permeability to molecules with diameters greater than ~4 Å, although no upper limit was defined [91]. These MDCK cells were protected from cytokine-induced increases in 3 kDa dextran flux [99]. Conversely, occludin overexpression augmented such cytokine-induced macromolecular flux [99]. However, the studies found that cytokine treatment paradoxically increased transepithelial electrical resistance (TER) in MDCK monolayers and that these TER increases were either attenuated or exaggerated by occludin knockdown or overexpression, respectively [99]. Subsequent studies of Caco-2 monolayers demonstrated the opposite, that occludin knockdown prevented TNF-induced TER loss [89]. TNF was also unable to increase paracellular macromolecular permeability of occludin knockdown Caco-2 monolayers. Thus, macromolecular permeability of TNF-treated, occludin-sufficient Caco-2 monolayers was similar to that of occludin-deficient monolayers regardless of TNF treatment [89]. The impact of occludin on barrier function required the C-terminal coiled-coil occludin/ELL domain (OCEL) domain, as barrier function of occludin-deficient Caco-2 monolayers was enhanced by EGFP-occludin, but not EGFP-occludin^ΔOCEL^, expression [89]. EGFP-occludin expression also restored TNF-sensitivity to occludin-knockdown Caco-2 monolayers, but monolayers expressing EGFP-occludin^ΔOCEL^ remained insensitive to TNF [89]. These data indicate that the same barrier defects are induced by genetic occludin knockdown or TNF-induced occludin removal from the tight junction [89]. Thus, while not essential for tight junction assembly, occludin is a critical regulator of the macromolecular, leak pathway barrier.

### 3.5. MLCK-Induced Occludin Endocytosis Requires ZO-1 Interactions with the Occludin OCEL Domain

Despite a close functional relationship, molecular sites of interactions between occludin and microfilaments have not been defined. It is, therefore, not clear how MLCK-mediated actomyosin contraction induces occludin endocytosis. One possibility is that ZO-1 acts as an intermediate that links occludin to F-actin. Although this has not been explored in detail, the free OCEL domain, which mediates occludin binding to ZO-1, does act as a dominant negative to prevent TNF-induced occludin endocytosis [89]. Moreover, occludin K433, located within the ZO-1 binding site, is critical to this dominant negative OCEL activity [89]. Thus, although further study is needed, ZO-1 may link occludin endocytosis to MLCK-dependent actomyosin contraction. This may also be related to tight junction-dependent mechanosensation, which involves ZO-1, ZO-2, the transcription factor DbpA/ZONAB, and their interactions with the cytoskeleton [100,101].

## 4. Regulation of MLCK Expression and Localization

### 4.1. Regulation of MLCK Transcription

The critical role of MLCK in acute, TNF-induced barrier loss was initially demonstrated in vitro using pharmacological inhibitors [50,54,55] and in vivo using a combination of pharmacological inhibitors and mice lacking epithelial long MLCK [51]. Further study demonstrated that, beyond MLCK enzymatic activity, transcriptional upregulation of long MLCK expression was essential to TNF-induced barrier loss [54]. TNF activated long MLCK transcription via the high-affinity TNF receptor TNFR2, thereby explaining how extremely low, nanogram concentrations of TNF were sufficient to trigger MLCK upregulation and barrier dysfunction in vitro [102]. Moreover, these data demonstrated that requirement for interferon-γ (IFN-γ) pretreatment before some cell monolayers were able to respond to TNF reflected IFN-γ-induced TNFR2 transcription [54,102]. Consistent with this, in vivo studies demonstrated that TNFR2 signaling was required for epithelial long MLCK upregulation during disease progression in T cell transfer-induced, experimental, chronic inflammatory bowel disease [103].

In vitro studies from two groups conflict as to whether TNF-induced MLCK upregulation depended on NFκB or AP-1 signaling [54,104]. One group found that the NFκB inhibitors curcumin, triptolide, and pyrrolidine dithiocarbamate prevented TNF-induced MLCK upregulation and barrier loss [104,105]. A second group found that a series of NFκB inhibitors, including MG132, capsaicin, curcumin, and triptolide were unable to prevent TNF-induced barrier loss [54]; MG132 and triptolide actually enhanced TNF-induced barrier loss. This group found that sulfasalazine was able to block TNF-induced barrier loss but did so in a dose dependent manner; barrier loss was prevented at 0.5 mM sulfasalazine but exaggerated at 2 mM sulfasalazine. Biochemical studies of NFκB RelA translocation to the nucleus and luciferase expression from an NFκB-responsive promoter showed that MG132 and 2 mM sulfasalazine, but not 0.5 mM sulfasalazine, inhibited TNF-induced NFκB activation. In contrast, 0.5 mM sulfasalazine, but not 2 mM sulfasalazine or MG132, blocked TNF-induced MLCK upregulation and MLC phosphorylation [54]. These data clearly separated NFκB from MLCK upregulation and suggested that another signaling pathway activated by TNF was responsible for barrier loss. Although both of these groups used Caco-2 cells in their studies, one possible explanation could be that the first group used a relatively undifferentiated Caco-2 clone while the second group used the differentiated, enterocyte-like Caco-2_BBe_ subclone [106].

Both groups went on to clone the human long MLCK promoter, which contained functional binding sites for both NFκB and AP-1 [107,108]. However, results again differed between the groups. The first group used a human genome database search to identify the 2 kb upstream of the human long MLCK transcriptional start site and eight NFκB binding sites within that region [104,107]. They found that two of these were active, with one repressing and the other increasing transcription ~2-fold [104]. In contrast, the second group used 5′ Rapid Amplification of cDNA Ends (5′-RACE) to identify two different long MLCK transcriptional start sites [108]. The upstream 4 kb sequence contained three functional AP-1 sites and two functional NFκB sites, all of which could regulate long MLCK transcription [108]. However, the intact 4 kb long MLCK promoter was not responsive to TNF prior to 14 days after confluence, suggesting that Caco-2 differentiation, which occurs progressively in the 2 weeks following growth to confluence, was required for full promoter activation [108]. From 3 to 14 days after confluence, activity of the MLCK promoter in response to TNF increased ~7-fold and transcription from an AP-1-responsive promoter increased ~2-fold, but transcription from an NFκB -responsive promoter decreased ~2-fold. Taken as a whole, these data suggest a unified model that explains the data from both groups. In this model, undifferentiated intestinal epithelial cells modestly upregulate long MLCK via NFκB signaling, while TNF induces more extensive MLCK upregulation via AP-1 signaling within differentiated cells. Consistent with this, the first group has gone on to show that AP-1-activating elements, such as mitogen activated protein (MAP) kinases, are involved in cytokine-induced MLCK upregulation [109,110].

### 4.2. MLCK Expression in Chronic Intestinal Disease

Quantitative morphometry of biopsies from human patients showed that MLCK expression and activity were increased in ulcerative colitis and Crohn’s disease [111]. Consistent with a role for TNF signaling, the magnitude of MLCK activation correlated directly with the degree of inflammatory activity in these biopsies.

Further analyses of the specific contributions of MLCK activation to disease were performed using transgenic mice expressing constitutively-active MLCK within the intestinal epithelium or global long MLCK knockout mice. Mice expressing constitutively-active MLCK displayed increased intestinal paracellular leak pathway permeability and increased MLC phosphorylation relative to non-transgenic littermates [75]. Both of these could be corrected by enzymatic MLCK inhibition [75]. Although these barrier defects were insufficient to induce spontaneous disease, compensatory mucosal immune activation including increased lamina propria T cell numbers, mild Th1 polarization, and heightened acute responses to infectious pathogens were detected [75,112]. However, when studied using the T cell transfer model of chronic inflammatory bowel disease, the transgenic mice developed disease more rapidly than non-transgenic littermates [75]. Moreover, overall disease severity was greater in transgenic mice and survival was markedly reduced. Thus, MLCK upregulation can impact progression of experimental inflammatory bowel disease.

Conversely, global long MLCK knockout mice were markedly protected from T cell transfer-induced colitis [103]. Although reduced disease could, potentially, reflect deletion of long MLCK in cells other than intestinal epithelium, the protection afforded by long MLCK knockout could be overcome by intestinal epithelial-specific constitutively-active MLCK expression [75]. Thus, MLCK inhibition is a potential target in chronic inflammatory bowel disease. It should, however, be noted that MLCK has many functions beyond tight junction regulation. For example, MLCK activation is critical to epithelial migration and wound repair [113,114,115]. Consistent with this, long MLCK knockout mice fared worse than their wild type counterparts when subjected to dextran sulfate sodium (DSS)-induced epithelial damage [103]. Similarly, in vivo knockout of nonmuscle myosin II markedly disrupts intestinal homeostasis, as demonstrated by goblet cell and barrier loss; as expected, these severely compromised mice are hypersensitive to DSS-induced injury [116]. Finally, MLCK loss may also have other consequences, either directly or as a secondary effect following disruption of cytoskeletal organization, protein trafficking, and signal transduction [117]. Thus, while intestinal epithelial MLCK inhibition and tight junction barrier preservation may be helpful in immune-mediated disease, they may also be associated with toxicities when epithelial damage predominates.

### 4.3. Enzymatic MLCK Inhibition is Not Feasible as a Therapeutic Intervention

**Figure 3:**
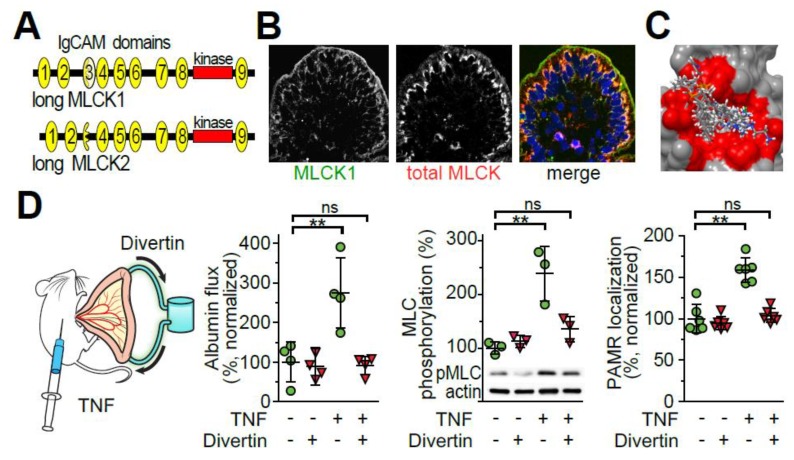
Specific targeting of long MLCK isoform 1 (MLCK1) prevents TNF-induced barrier loss in vivo. (**A**) Protein domain structure of long MLCK isoforms 1 and 2. Immunoglobulin-cell adhesion molecule (IgCAM) domains are numbered from the amino terminus. (**B**) Long MLCK1 (green), total MLCK (red), and nuclei (blue) in normal human jejunum. MLCK1 is preferentially-localized to the perijunctional actomyosin ring. (**C**) Virtually screened compounds docked to a binding pocket within the unique IgCAM3 domain of MLCK1. (**D**) Mice were injected with vehicle or TNF, and jejunal loops were perfused with either saline-containing vehicle or Divertin. TNF-induced increases in albumin flux (from bloodstream into the gut lumen) were blocked by Divertin. Divertin also blocked TNF-induced myosin II regulatory light chain (MLC) phosphorylation and MLCK1 recruitment to the PAMR. Notably, Divertin does not inhibit MLCK enzymatic activity. ** *p* < 0.01 by ANOVA with Dunn’s multiple comparison test. From Graham et al. *Nat. Med.* 2019.

Three distinct MLCK genes are present in mammals. These are *MYLK*, the smooth muscle/non-muscle MLCK located on human chromosome 3; skeletal muscle *MYLK2* on human chromosome 20; and cardiac *MYLK3* on human chromosome 16. *MYLK* encodes a long MLCK expressed in intestinal epithelium as well as short MLCK and telokin [118,119]. Telokin has regulatory functions but cannot phosphorylate MLC [120,121,122]. In contrast, both short MLCK, an ~110-130 kDa protein expressed in smooth muscle, and long MLCK, an ~215 kDa protein expressed in non-muscle cells, including epithelium, share identical catalytic and calmodulin-binding regulatory domains (Figure 3A). Thus, neither chemical nor genetic inhibitors can distinguish between short and long MLCK.

Enzymatic MLCK inhibition could not be used therapeutically since, as noted above, long and short MLCK have identical catalytic domains. In mice, short MLCK knockout leads to death in the early perinatal period [123]. Development of inducible knockout mice with loxP sites flanking the *MYLK* sequence encoding the catalytic domain overcame this limitation [124]. However, tissue-specific knockout in smooth muscle resulted in hypotension, bladder dysfunction, and severe intestinal dysmotility, and death [124]. Thus, while targeted inhibition of MLCK-mediated tight junction barrier regulation might have therapeutic benefit, loss of MLCK enzymatic activity in smooth muscle [124] and non-muscle [103,113] cells would have unacceptable toxicities.

### 4.4. TNF Induces IgCAM3-Mediated Long MLCK1 Recruitment to the Perijunctional Actomyosin Ring

The difference between long and short MLCK is the presence of a long stretch of N-terminal sequence in long MLCK [125,126,127]. The exons that encode that part of the protein undergo extensive alternative splicing, resulting in a number of different long MLCK splice variants [127]. Only isoforms MLCK1 and MLCK2, which differ by a single 207 nucleotide exon, are expressed in intestinal epithelia [125]. The differential functions of these and other long MLCK splice variants are not well-defined. However, it has been reported that the 69 amino acids encoded by the 207 nucleotide exon that distinguishes MLCK1 from MLCK2 include a src kinase target site whose phosphorylation can regulate MLCK activity [126].

Tissue analysis using an antibody specific for long MLCK1 demonstrated that, relative to total epithelial long MLCK, long MLCK1 was concentrated within the perijunctional actomyosin ring [125] (Figure 3B). Moreover, targeted knockdown of long MLCK1 within Caco-2 monolayers reduced paracellular permeability [125]. Further study showed that, in addition to increasing long MLCK expression, TNF specifically induced long MLCK1 recruitment to the perijunctional actomyosin ring [128]. This selective effect on MLCK1 was only post-translational, as intestinal epithelial MLCK1 and MLCK2 transcripts were comparably increased by TNF (unpublished data, Graham and Turner).

Structural analysis indicated that the 69 amino acids unique to long MLCK1 completed the immunoglobulin-cell adhesion molecule (IgCAM) domain 3, one of nine IgCAM domains within long MLCK1 [128]. Because this is the only difference between MLCK1 and MLCK2, it stands to reason that the key features responsible for TNF-induced MLCK 1 recruitment to the perijunctional actomyosin ring reside within IgCAM3. Consistent with this, expression of the IgCAM3 domain alone attenuated TNF-induced MLCK1 recruitment to the perijunctional actomyosin ring and barrier regulation, suggesting that it disrupted interactions between MLCK1 and other intracellular molecules (unpublished data, He and Turner).

### 4.5. TNF Induces IgCAM3-Mediated Long MLCK1 Recruitment to the Perijunctional Actomyosin Ring

The observation that an enzymatically inactive region, i.e., IgCAM3, was responsible for long MLCK1 recruitment to the perijunctional actomyosin ring presented an opportunity for blocking MLCK-dependent tight junction regulation in disease. After solving the IgCAM3 crystal structure [128,129], a hydrophobic drug-binding pocket within IgCAM3, but not the other long MLCK1 IgCAM domains, was identified. Importantly, this drug binding pocket was conserved between human and mouse long MLCK1 [128]. An in silico screen using molecular docking software identified a small number of candidate molecules with putative binding to the targeted hydrophobic pocket (Figure 3C). In vitro screening demonstrated that one of these was able to increase intestinal epithelial barrier function without inhibiting MLCK enzymatic activity in a cell-free assay, disrupting smooth muscle contraction, or interfering with epithelial wound repair, i.e., migration. This molecule, termed Divertin, was, however, able to displace long MLCK1 from the perijunctional actomyosin ring, reverse TNF-induced barrier loss, and reduce MLC phosphorylation in Caco-2 monolayers [128].

Consistent with its targeted effect on MLCK1-dependent tight junction regulation, mice could receive daily Divertin administration for 30 days without any apparent toxicity. Divertin was, however, able to prevent MLCK1 recruitment to the perijunctional actomyosin ring, MLC phosphorylation, occludin internalization, and leak pathway barrier loss in response to acute TNF challenge in mice (Figure 3D). Divertin also prevented perijunctional MLCK1 recruitment, MLC phosphorylation, and occludin internalization in human intestinal biopsies treated with TNF in vitro. Thus, Divertin was able to reverse and prevent acute, TNF-induced MLC phosphorylation and barrier loss in vitro and in vivo [128] (Figure 3D).

**Figure 4:**
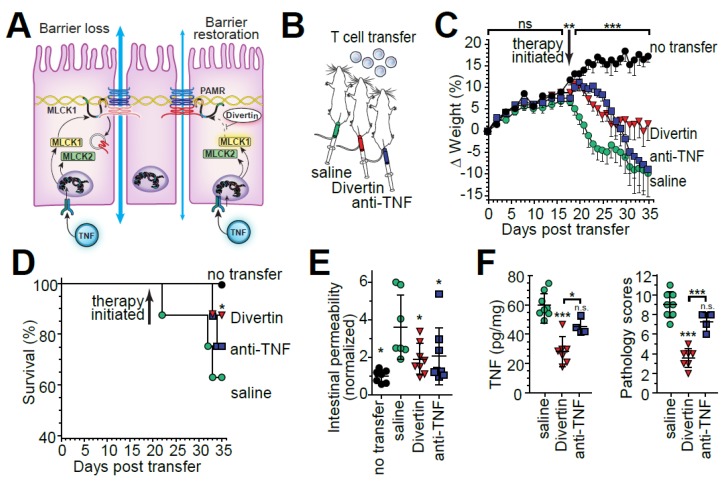
Inhibition of MLCK1 recruitment to the perijunctional actomyosin ring attenuates immune-mediated colitis. (**A**) Proposed mechanism of Divertin action. (**B**) Immunodeficient mice received naïve CD4+ effector T cells. Therapy with saline (vehicle), divertin, anti-TNF, or combined Divertin and anti-TNF was initiated after definitive features of disease developed (day 19). (**C**) Divertin limited weight loss after T cell transfer and was superior to anti-TNF antibody treatment. ** *p* < 0.01 by two-tailed t test for no transfer vs. all other mice at day 18. *** *p* < 0.001 by ANOVA with Tukey’s multiple comparison test over the interval from 19−35 days. (**D**) Divertin enhanced survival after T cell transfer. * *p* < 0.05, versus saline-treated mice, by Gehan–Breslow–Wilcoxon test. (**E**) Divertin limited intestinal barrier loss. * *p* < 0.05, versus saline-treated mice, by ANOVA with Newman–Keuls multiple comparison test. (**F**) Divertin was superior to anti-TNF antibody treatment in limiting mucosal cytokine production and histopathology after T cell transfer. * *p* < 0.05; *** *p* < 0.001 by ANOVA with Bonferroni correction. From Graham et al. *Nat. Med.* 2019.

The efficacy of Divertin and restoring intestinal barrier function in chronic disease was initially assessed using IL-10 knockout mice, which develop in intestinal barrier defect early in the course of disease [128,130]. Although Divertin did not affect intestinal permeability in wild type mice, it was able to restore the intestinal barrier in IL-10 knockout mice [128]. Moreover, Divertin was markedly effective in limiting disease during T cell transfer colitis regardless of whether it was administered just prior to or after clinical disease presentation [128] (Figure 4). Remarkably, all measures assessed showed that Divertin was superior or equivalent to anti-TNF in limiting T cell transfer colitis severity [128] (Figure 4). Some data did, however, indicate that the beneficial effects of Divertin might be additive to, or even synergistic with, those of anti-TNF. Thus, preventing MLCK1 recruitment to the perijunctional actomyosin ring may be a non-toxic approach to limiting or preventing immune-mediated colitis either alone or in combination with immunomodulatory agents, e.g., anti-TNF [128] (Figure 4).

## 5. Perspective and Future Directions

The past 25 years have seen tremendous growth in our understanding of tight junction structure, cell biology, and pathobiology. Most recently, this has resulted in the develop of a proof-of-concept molecule that may provide a foundation for creation of actual therapeutic agents. Despite the enthusiasm this has engendered, there remains much to be learned. Specific topics include elucidation of mechanisms that regulate MLCK1 recruitment to the perijunctional actomyosin ring, further definition of pore pathway function in health and disease, and characterization of the structural and functional properties of defined and to-be-discovered tight junction components.
